# Case Report: Bilateral Spigelian Hernia With Meckel’s diverticulum

**DOI:** 10.12688/f1000research.157529.1

**Published:** 2024-11-11

**Authors:** Younis Mohamed, Ahmed Hussein, Baber Chaudhary, Omar Elsaba, Mahmoud Rhodes

**Affiliations:** 1 General Surgery, NHS Wales Betsi Cadwaladr University Health Board, Bangor, Wales, LL572LH, UK; 2 Trauma and Orthopedic, NHS Wales Betsi Cadwaladr University Health Board, Bangor, Wales, LL57 2PW, UK; 3 General Surgery, NHS Wales Betsi Cadwaladr University Health Board, Bangor, Wales, LL57 2PW, UK; 4 Urology Specialist Doctor, BCUHB, Wales, UK; 5 MRCS, Trauma and Orthopaedic Registrar, Nasser institute Hospital, Cairo, Egypt

**Keywords:** #meckel´s diverticulum, #spigelian hernia, #emergency exploration, #bilateral spigelian hernia, #acute abdomen

## Abstract

Spigelian hernia is an uncommon form of ventral hernia, with an incidence ranging from 0.1% to 2%. This case report describes a 72-year-old female who presented with an obstructed right Spigelian hernia, a left Spigelian hernia, and an uncomplicated Meckel’s diverticulum, along with the management approach. The patient had experienced intermittent tenderness in the right iliac fossa for the last two months, which had worsened to severe pain over the previous two days, accompanied by a palpable mass in the right lower quadrant. An urgent CT scan of the abdomen and pelvis revealed an obstructed right Spigelian hernia containing dilated proximal small bowel, and a left uncomplicated spigelian hernia. The patient experienced worsening abdominal pain and vomiting. Emergency laparotomy was performed, revealing a right Spigelian hernia with viable small bowel loops, a non-complicated Meckel’s diverticulum located 20 cm from the ileocecal valve, and a small left Spigelian hernia. The right Spigelian hernia was repaired using intraperitoneal sublay mesh, while the left hernia was treated with primary repair. No bowel resection was performed at the site of the Meckel’s diverticulum, as it was non-inflamed, to prevent contamination of the mesh with bowel flora. Bilateral Spigelian hernias accompanied by Meckel’s diverticulum present a challenging clinical scenario. Although rare, this condition should be considered in the differential diagnosis of acute abdominal pain due to the potential for serious complications.

## Introduction

Spigelian hernia is a rare form of ventral hernia. It involves the protrusion of preperitoneal fat, peritoneal sac, or intra-abdominal organs through a defect in the Spigelian aponeurosis, which lies between the rectus abdominis muscle medially and the semilunar line laterally. The Spigelian aponeurosis, formed by the fusion of the internal oblique and transversus abdominis aponeuroses, constitutes a structurally vulnerable region of the abdominal wall, predisposing it to herniation under conditions of elevated intra-abdominal pressure. Predisposing factors include obesity, chronic obstructive pulmonary disease (COPD), prior abdominal surgery, and trauma. While most Spigelian hernias are acquired, congenital forms have also been reported, potentially due to embryological defects in the development of the abdominal musculature. ^1^’

Spigelian hernias are challenging to diagnose clinically because the defect is often masked by the overlying external oblique aponeurosis, making it difficult to detect on physical examination. As a result, patients may present acutely with complications such as incarceration, strangulation, or bowel obstruction. Given the high risk of these complications, Spigelian hernias are not suitable for conservative management, and surgical intervention is the recommended treatment in both elective and emergency cases. ^2^

This case report presents a patient with an obstructed right lower quadrant Spigelian hernia, as well as a left-sided Spigelian hernia accompanied by a non-complicated Meckel’s diverticulum, which was successfully managed through open laparotomy.

## Case presentation

A 72-year-old female with a history of bronchial asthma, obesity, and left-sided breast cancer treated with mastectomy, currently on Anastrozole therapy, in addition to anxiety and depression history, presented to the emergency department with progressively worsening right iliac fossa pain over the past two days. The pain had been intermittent for two months. She also reported the presence of a palpable mass in the right lower quadrant, accompanied by nausea and multiple episodes of vomiting the previous night. Her bowel habits were normal, and there was no per-rectal bleeding.

**Figure 1.**
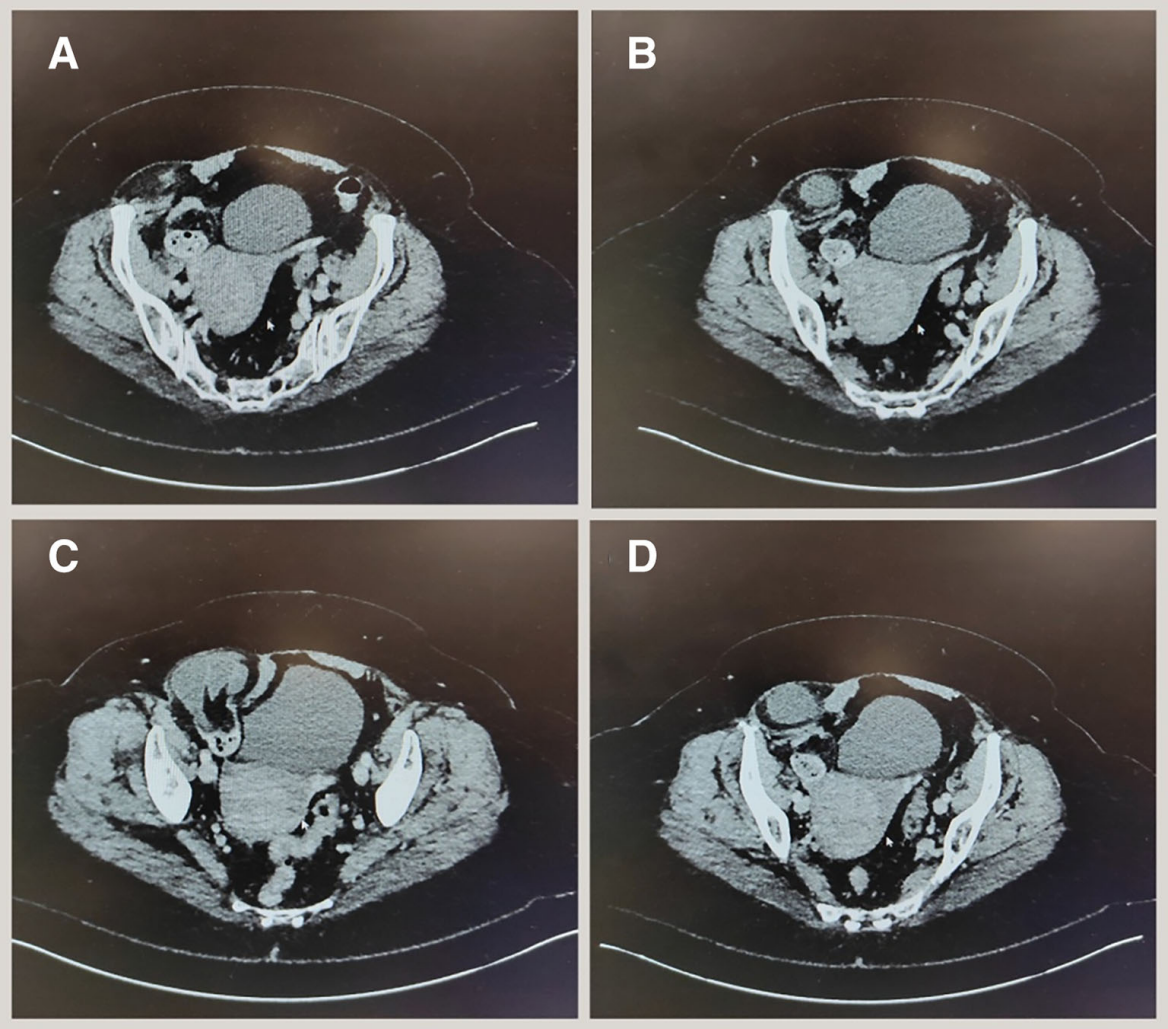
Right spigelian hernia most prominent in image C and small spigelian defect noted at the left side without hernia sac noted in image B.

On physical examination, significant tenderness was noted in the right iliac fossa, and a firm mass was palpated along the lateral border of the rectus abdominis muscle, approximately midway between the umbilicus and the symphysis pubis. The mass became more prominent when the patient was examined in a standing position and was associated with voluntary guarding. Symptomatic relief was provided with analgesics and antiemetics. Laboratory investigations revealed a white cell count of 9,700 WBC/μL, hemoglobin of 146 g/dL, platelet count of 366,000 platelets/μL. A contrast-enhanced CT scan of the abdomen and pelvis ( Figure 1) identified a right-sided Spigelian hernia containing small bowel loops, with proximal small bowel dilatation suggestive of developing obstruction, with incidental findings included a simple liver cyst and a bulky uterus. After discussing the surgical options, the patient consented to proceed with an open laparotomy.

Preoperative preparation involved keeping the patient nil by mouth (NBM), administering intravenous fluids (IVF), obtaining an electrocardiogram (ECG), scheduling the operating room, securing informed consent, performing a blood group and save, administering symptomatic medications, and conducting an anesthetic review. The procedure was performed under general and local anesthesia, with prophylactic intravenous antibiotics administered within one hour prior to the incision.

**Figure 2.**
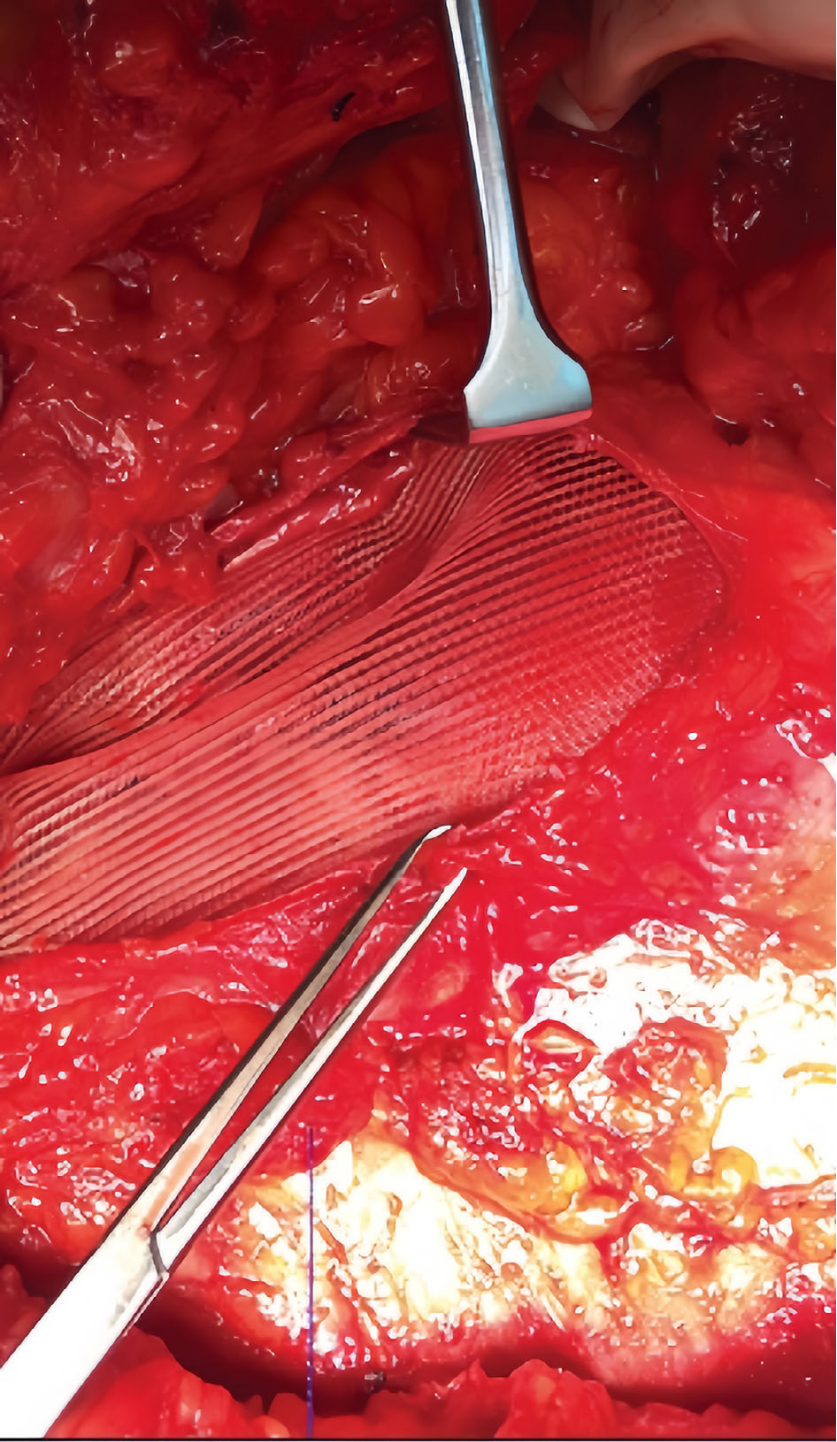
Sublay Mesh Repair (Retromuscular/Subfascial) **:** The mesh is placed between the right rectus abdominis muscle and posterior rectus sheath.

During surgery, a lower midline incision was made. A Meckel’s diverticulum was identified approximately 20 cm from the ileocecal valve. Although bruised, it was not inflamed, so it was left intact to avoid contamination of the mesh with bowel flora. Bilateral hernial defects were observed, with the right hernia being larger than the left. Both defects were located adjacent to the lateral rectus sheath, and the right Spigelian hernia contained small bowel loops and preperitoneal fat. The small bowel loops were examined up to the duodenojejunal (DJ) junction, with no abnormalities detected. The right Spigelian hernial sac was opened, and its contents were inspected. The hernia defect was closed from the peritoneal side using 2-0 Vicryl sutures, and a 6×11 cm Prolene mesh was placed in a sublay position deep to the rectus muscle. The left Spigelian hernia was repaired using primary suture closure with 2-0 Prolene. A transversus abdominis plane (TAP) block was administered by the anesthetist. Postoperatively, the patient was advised to resume oral intake and continue antibiotic therapy for 48 hours ( Figure 2).

**Figure 3.**
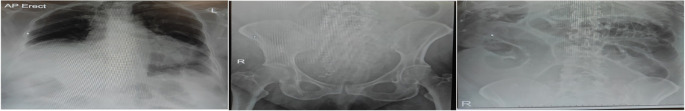
X-ray chest and abdomen showing atelctasis with dilated small bowel loops.

On postoperative day 1, the patient experienced moderate abdominal pain, multiple episodes of greenish vomiting, with absence of bowel movements, abdominal bloating, and reduced urine output of less than 30 mL/hour. Intravenous fluids were administered, and a nasogastric tube (NGT) and urinary catheter were inserted. Oral intake was limited to sips of water. A chest and abdominal X-ray revealed atelectasis and dilated small bowel loops ( Figure 3).

**Figure 4.**
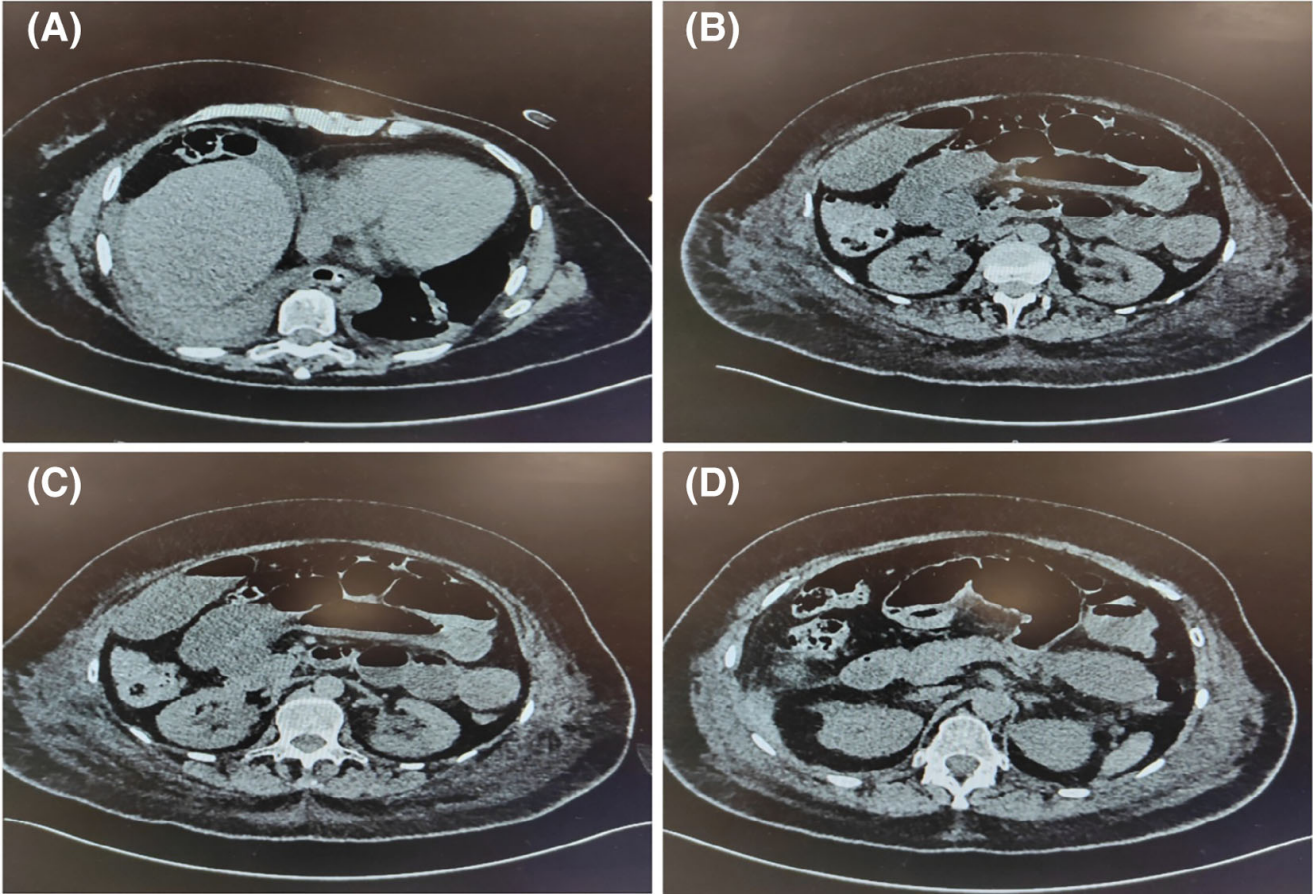
multiple loops of distended small intestine which contain an air-fluid level, represented post operative ileus.

By postoperative day 2, the patient’s symptoms persisted, and blood tests showed elevated inflammatory markers: CRP of 246 mg/L, WCC of 11.5 × 10 ^9^/L, hemoglobin of 122 g/L, and platelet count of 358 × 10 ^9^/L. Due to concern for an intraperitoneal collection or bowel perforation, a CT abdomen and pelvis (CTAP) was performed, revealing multiple distended small bowel loops with air-fluid levels, consistent with postoperative ileus ( Figure 4).

By postoperative day 3, the patient passed a bowel movement and began mobilizing with physiotherapy assistance. Initially, she did not meet the physiotherapy assessment criteria, but her physical performance gradually improved in the following days. By postoperative day 6, she was able to mobilize independently and was discharged home. A follow-up appointment was scheduled for six weeks later.

## Discussion

Spigelian hernia is a rare abdominal wall defect, with its incidence peaking between the 4th and 7th decades of life. A slight female predilection has been noted, with a male-to-female ratio of 1:1.183. The hernia occurs within the “Spigelian belt,” a 6 cm wide zone along the Spigelian line extending caudally from the umbilicus to the inferior epigastric vessels. Based on the relationship to these vessels, Spigelian hernias are classified into two types: high hernias (cranial to the vessels) and low hernias (caudal to the vessels, within Hesselbach’s triangle). ^3^

**Figure 5.**
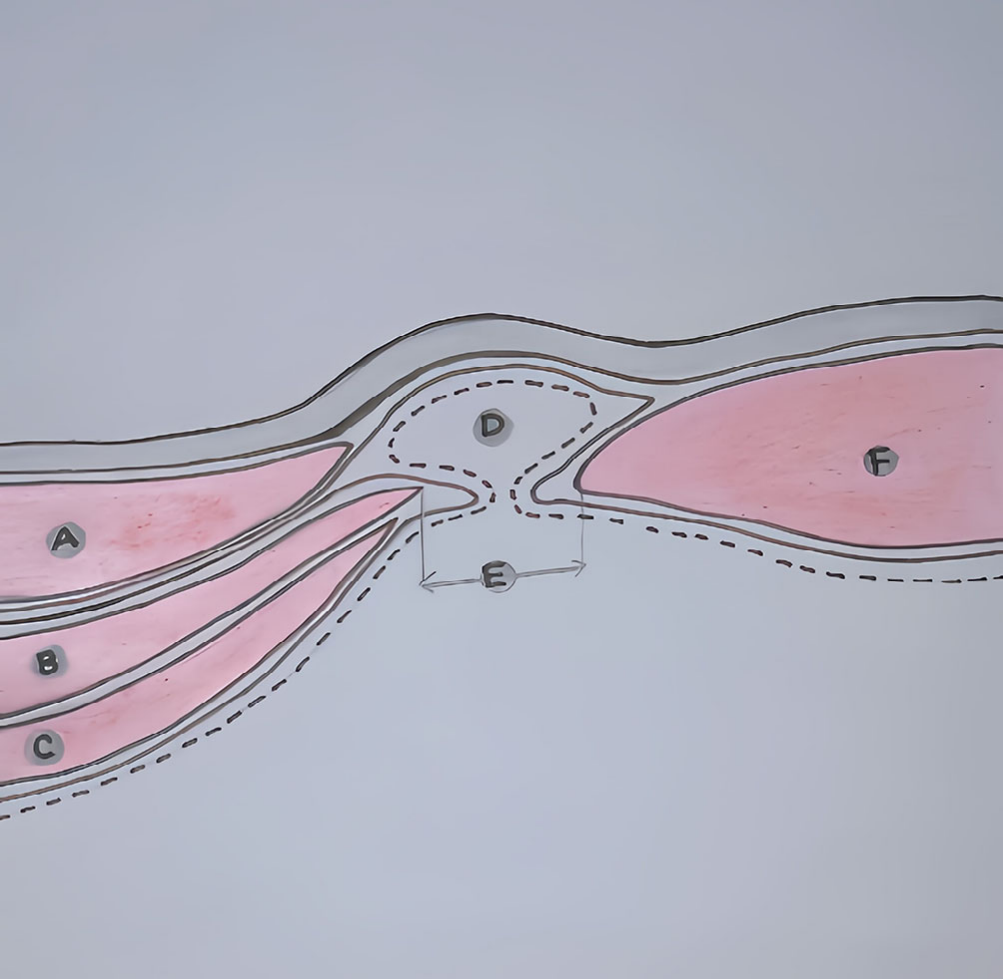
Rendering of a Spigelian hernia through the various tissue layers. A) External oblique. B) Internal oblique. C) Transverse abdominis. D) Hernia sac with contents. E) Spigelian fascia. F) Rectus abdominis.

Several theories explain the etiology of Spigelian hernias. Cooper’s vascular-nervous theory suggests weakening of the Spigelian belt due to the deep iliac branch of the inferior epigastric vessels penetrating the aponeurosis. Zimmerman’s musculo-aponeurotic fasciculation theory is more widely accepted, proposing that infiltration of preperitoneal fat weakens the deep fibrous musculature, predisposing it to herniation ( Figure 5). Other theories, such as the embryologic transition theory and Watson and Iason’s theory, implicate areas of lower resistance between developing abdominal wall muscles and weaknesses at the junction of the semilunar line and arcuate line, respectively. Risk factors for Spigelian hernia include obesity, chronic obstructive pulmonary disease (COPD), prior abdominal surgery, trauma, and conditions increasing intra-abdominal pressure. ^4^

Clinically, Spigelian hernias often evade early detection due to their deep location beneath the external oblique aponeurosis, making physical examination findings subtle or absent. Early symptoms can be nonspecific, ranging from vague abdominal discomfort to a palpable lump. However, the risk of complications, including incarceration (reported in 24–27% of cases) and strangulation (2–14%), is significant. These risks arise from the formation of a “rigid neck” in the hernial sac, particularly due to the Spigelian fascia’s fibrous bands. In this patient, morbid obesity and chronic cough associated with asthma likely contributed to the hernia formation, and she presented with a tender right iliac fossa mass, which was confirmed on CT imaging to be an obstructed Spigelian hernia, a situation warranting urgent surgical intervention. ^5^

Imaging is critical in diagnosing Spigelian hernias, especially given their frequent misdiagnosis in early stages. Computed tomography (CT) is the preferred modality for identifying the defect, its size, and contents, for appropriate surgical planning. In this case, CT identified the hernia and confirmed small bowel involvement, indicating impending obstruction and urgent surgical management. While physical examination alone often fails to detect Spigelian hernias, imaging techniques such as ultrasound and CT are crucial for early diagnosis and surgical planning. ^6^

Surgical intervention is the definitive treatment for Spigelian hernia, with both open and laparoscopic approaches offering these options based on patient factors, hernia characteristics, available resources, and the surgeon’s expertise. Laparoscopic repair is gaining popularity due to its advantages of better bilateral visualization, detection of contralateral defects, and shorter recovery times. However, open repair offers superior intra-operative direct visualization and dissection, particularly for larger or more complex hernias. Both approaches aim for complete defect closure, typically reinforced with mesh to reduce the risk of hernia recurrence. ^7^

Mesh reinforcement is strongly recommended in both open and laparoscopic spigelian hernia repairs, as outlined by the European Hernia Society (EHS) guidelines. Various mesh techniques exist, each selected based on the hernia size and complexity ^8^:

1. **Primary Suture Repair**: Suitable for small hernias, it involves closing the defect with non-absorbable sutures. However, it carries a higher recurrence risk for larger hernias.
2. **Onlay Mesh Repair**: A synthetic mesh is placed on the anterior rectus sheath to reinforce the defect, but it may cause complications such as seroma or wound infection.
3. **Sublay Mesh Repair (Retromuscular/Subfascial)**: Mesh is placed between the rectus muscle and posterior rectus sheath. This technique provides robust support with fewer complications, making it ideal for complex or recurrent hernias.
4. **Inlay Mesh Repair**: Mesh is positioned directly within the defect, offering simplicity but a higher recurrence risk due to incomplete coverage.
5. **Preperitoneal Mesh Repair**: Mesh placement in the preperitoneal space reduces mesh-related complications and is less invasive but can be technically demanding.
6. **Component Separation Technique**: This involves mobilizing abdominal muscles to close large defects without tension, potentially with mesh reinforcement. It allows closure of large defects but is more invasive and associated with higher wound complication risks.

In this case, the patient underwent open sublay mesh repair for the right-sided Spigelian hernia, while the left-sided hernia was repaired with primary sutures. This approach allowed for thorough intraoperative inspection of the hernia contents and effective defect closure. Although the recovery was prolonged by postoperative ileus, the patient showed gradual improvement and was successfully discharged.

Ultimately, the choice between open and laparoscopic repair depends on the hernia’s complexity and the surgeon’s expertise. Both approaches, when paired with mesh reinforcement, offer effective and durable repair modalities, with laparoscopic techniques gaining favor for their minimally invasive nature and faster recovery times.

## Conclusion

Spigelian hernia often presents a diagnostic challenge due to its lack of distinct physical findings, leading to frequent delays in diagnosis. A high index of clinical suspicion is essential, given the potential for complications such as bowel obstruction and strangulation. Fortunately, in this case, the patient presented with clear symptoms, allowing for timely surgical intervention and successful repair. Computed tomography (CT) imaging played a crucial role in confirming the diagnosis, and in instances where imaging is inconclusive, open laparotomy provides superior visualization and access to the peritoneal cavity, facilitating thorough inspection, especially in complex cases involving the small bowel and Meckel’s diverticulum.

Surgical intervention remains the cornerstone of treatment for Spigelian hernia, as supported by the literature. In this case, open repair with a Sublay Mesh technique was performed, placing the mesh between the rectus abdominis muscle and the posterior rectus sheath to reinforce the defect. While Spigelian hernias are rare, their high risk for acute complications necessitates that they remain a differential diagnosis in patients presenting with abdominal pain. Early recognition and prompt surgical management are key to preventing adverse outcomes.

## Consent for publication

A written informed consent for publication was obtained from the patient and the co-authors for this case report, including the publication of any identifying data related to the clinical history, images, and findings. The patient has provided informed written consent, acknowledging the inclusion of relevant medical information for educational and research purposes. All efforts were made to ensure that the patient’s identity remains protected and anonymized throughout the report. This consent is in accordance with the ethical guidelines and institutional policies governing the protection of patient privacy and confidentiality.

## Data Availability

All data underlying the results are available as part of the article and no additional source data are required.
