# Supplementary material for: Case Report: Bilateral Spigelian Hernia With Meckel’s diverticulum
Source: F1000Res. 2024 Nov 11;13:1352. [Version 1] doi: 10.12688/f1000research.157529.1 (PMC11624431; doi:10.12688/f1000research.157529.1)
Supplement: Peer review history

# Reviewer report: Reviewer response for version 1

**DOI:** 10.5256/f1000research.172986.r340365

**Published:** 2024-12-06

**Authors:** Barbara Yordanis Hernandez Cervantes

**Affiliations:** 1 Department of Surgery, School of Medicine, University of Health and Allied Sciences, Ho, Ghana

The case is well organized and complies with the standards for case reports. Nonetheless, I like to emphasize some places where I believe enhancements are essential for optimal comprehension of the case and for educational objectives.

 Certain grammatical errors in the text and image labels require correction.

 The authors stated in the abstract that the mesh was placed intraperitoneally, which contradicts the case description.

 In the case presentation, it is essential to comprehend the history of reducibility concerning the right swelling during the last two months of intermittent abdominal pain. This information would assist in determining whether we were addressing an incarcerated hernia or a reducible hernia. Furthermore, the narrative fails to address the reducibility of the mass during the two days of exacerbated discomfort, prompting me to conclude that it was irreducible.

 The physical examination lacks details regarding the left abdominal swelling, if present, and does not provide information on the assessment of the remaining abdominal quadrants, as bowel sounds are not noted as a significant indicator of intestinal obstruction.

 During the research, I noted that an accessible and effective method, such as abdominopelvic ultrasonography, was not employed (for what reason?).

 The surgical treatment does not clarify whether the Meckel’s diverticulum was included in the hernia sac; it is noted that the diverticulum exhibited bruising (what was the cause of this?). A visual representation is absent; however, it would benefit the surgical community, particularly since the decision was made not to resect the diverticulum, acknowledging that potential complications such as inflammation, bleeding, and obstruction may arise in the future.

 Due to the patient's paralytic ileus in the postoperative phase, a serum electrolyte test may be conducted, as it plays a crucial part in the condition's pathogenesis.

 This case report pertains to Spigelian hernias with Meckel's diverticulum. Nonetheless, I did not locate any information pertaining to the correlation and care of Meckel's diverticulum and hernias during the discussion of the case.

 Aside from two references, the remainder are over a decade old.

 Conclusions: In addition to these particulars, the case report is highly engaging and informative for the entire medical community, particularly the surgical sector.

Are enough details provided of any physical examination and diagnostic tests, treatment given and outcomes?

Yes

Is the case presented with sufficient detail to be useful for other practitioners?

Partly

Is sufficient discussion included of the importance of the findings and their relevance to future understanding of disease processes, diagnosis or treatment?

Partly

Is the background of the case’s history and progression described in sufficient detail?

Yes

Reviewer Expertise:

As a general surgeon, my scope of expertise encompasses a wide range of procedures, particularly abdominal surgery, with a focus on abdominal wall hernias, which represent one of the most prevalent diseases in our specialty globally.

I confirm that I have read this submission and believe that I have an appropriate level of expertise to confirm that it is of an acceptable scientific standard.

# Reviewer report: Reviewer response for version 1

**DOI:** 10.5256/f1000research.172986.r340369

**Published:** 2024-11-19

**Authors:** Atif Iqbal, Dr Hamid UR Rehman

**Affiliations:** 1 Department of General Surgery, Jinnah International Hospital, Abbottabad, Pakistan; 2 Surgery, Jinnah International Hospital, Abbottabad, Abbottabad., KPK, Pakistan

The article describes a 72-year-old female presenting with bilateral Spigelian hernias & an uncomplicated Meckel's diverticulum. Key clinical details include:

 Symptoms, Diagnostics, Management and Outcome.

**Strengths:**

 1. Comprehensive Case Description: Detailed reporting of the patient's history, clinical findings, diagnostic imaging, and treatment 

 2 Visual Aids: High quality imaging and diagrams provide clarity on the surgical approach

 3 Detailed Discussion: Thoroughly examines the rarity of the condition , challenges in diagnosis and its importance in acute abdominal pain differential diagnosis.

 4 Well constructed case report.

**Areas for Improvement**

 There is always room at the top. Article is well written and comprehensive. If authors could mention BMI of the patient and urea/creatinine/electrolytes results, article will be almost perfect.

**Conclusion:**

 The case report is a valuable addition to medical literature, offering insights into a rare condition and its management.

**Approval Status: Approved**

Are enough details provided of any physical examination and diagnostic tests, treatment given and outcomes?

Yes

Is the case presented with sufficient detail to be useful for other practitioners?

Yes

Is sufficient discussion included of the importance of the findings and their relevance to future understanding of disease processes, diagnosis or treatment?

Yes

Is the background of the case’s history and progression described in sufficient detail?

Yes

Reviewer Expertise:

General surgery articles

We confirm that we have read this submission and believe that we have an appropriate level of expertise to confirm that it is of an acceptable scientific standard.
